# CtBP Neuroprotective Role in Toxin-Based Parkinson’s Disease Models: From Expression Pattern to Dopaminergic Survival

**DOI:** 10.1007/s12035-023-03331-w

**Published:** 2023-04-15

**Authors:** Cláudia Saraiva, Jéssica Lopes-Nunes, Marta Esteves, Tiago Santos, Ana Vale, Ana Clara Cristóvão, Raquel Ferreira, Liliana Bernardino

**Affiliations:** 1 Brain Repair Group, Health Sciences Research Center (CICS-UBI), Faculty of Health Sciences, University of Beira Interior, Av. Infante D. Henrique, 6200-506 Covilhã, Portugal; 2 Present Address: Luxembourg Centre for Systems Biomedicine (LCSB), University of Luxembourg, 7 Avenue Des Hauts-Fourneaux, Esch-Sur-Alzette, Luxembourg; 3 Present Address: CEDOC, NOVA Medical School|Faculdade de Ciências Médicas, Universidade NOVA de Lisboa, Campo Dos Mártires da Pátria, 130, Lisboa, Portugal

**Keywords:** Parkinson’s disease, C-terminal binding proteins, Neuroprotection, Dopaminergic survival, Aging

## Abstract

C-terminal binding proteins (CtBP) are transcriptional co-repressors regulating gene expression. CtBP promote neuronal survival through repression of pro-apoptotic genes, and may represent relevant targets for neurodegenerative disorders, such as Parkinson’s disease (PD). Nevertheless, evidence of the role of CtBP1 and CtBP2 in neurodegeneration are scarce. Herein, we showed that CtBP1 and CtBP2 are expressed in neurons, dopaminergic neurons, astrocytes, and microglia in the *substantia nigra* (SN) and striatum of adult mice. Old mice showed a lower expression of CtBP1 in the *SN* and higher expression of CtPB2 in the *SN* and striatum compared with adult mice. *In vivo* models for PD (paraquat, MPTP, 6-OHDA) showed increased expression of CtBP1 in the *SN* and striatum while CtBP2 expression was increased in the striatum of paraquat-treated rats only. Moreover, an increased expression of both CtBP was found in a dopaminergic cell line (N27) exposed to 6-OHDA. In the 6-OHDA PD model, we found a dual effect using an unspecific ligand of CtBP, the 4-methylthio 2-oxobutyric acid (MTOB): higher concentrations (e.g. 2500 µM, 1000 µM) inhibited dopaminergic survival, while at 250 μM it counteracted cell death. *In vitro*, this latter protective role was absent after the siRNA silencing of CtBP1 or CtBP2. Altogether, this is the first report exploring the cellular and regional expression pattern of CtBP in the nigrostriatal pathway and the neuroprotective role in PD toxin-based models. CtBP could counteract dopaminergic cell death in the 6-OHDA PD model and, therefore, CtBP function and therapeutic potential in PD should be further explored.

**Supplementary Information:**

The online version contains supplementary material available at 10.1007/s12035-023-03331-w.

## Introduction

C-terminal binding proteins (CtBP) are highly conserved proteins whose primary function is to repress transcription [1]. CtBP regulate gene expression by targeting various chromatin-modifying factors to the promoter-bound repressors. The CtBP corepressor complex mediates histone modifications by deacetylation and methylation [1]. In vertebrates, CtBP are encoded by two different genes, CtBP1 and CtBP2, and each produces different isoforms. CtBP share the conserved amino acid motif Pro-X-Asp-Leu-Ser (PXDLS) and the RRTGXPPXL (RRT motif) domains, which are essential to recruiting the core co-repressor machinery [2–4], and the dehydrogenase domain, which has an affinity for both nicotinamide adenine dinucleotide (NAD^+^) and nicotinamide adenine dinucleotide hydride (NADH) [5]. CtBP have environmental and metabolic sensing capability, exhibiting oxygen sensing and glycolysis-regulated transcriptional activities. CtBP perform both transcriptional and non-transcriptional functions. In fact, the long splice forms of CtBP proteins (CtBP1/2-L) have co-repression activity and are mainly localized in the nuclear compartment [6, 7]. Nevertheless, the smaller isoform of CtBP1 (CtBP1-S) is mainly cytosolic [7]. For example, CtBP1 has been described in synaptic ribbons of sensory neurons and pre-synaptic neuronal terminals [8–10] and reported to be important for membranal traffic and Golgi partitioning during mitosis [11]. Likewise, a CtBP2 isoform, called RIBEYE, has also been described as important for forming ribbon synapses of sensory neurons and bipolar cell development [12, 13].

Gene knockout of CtBP1 or CtBP2 in mice resulted in severe embryonic defects or lethality, demonstrating their crucial role during development. *CtBP1*-null mice were 30% smaller although viable, while *CtBP2*-null mice exhibit embryonic lethal phenotype, and different degrees of combined allele CtBP1 and CtBP2 mutations resulted in differential developmental defects, indicating that CtBP have both unique and overlapping functions [3]. CtBP2 has been linked to cortical development, with its overexpression in cortical cells causing impairments in the migration of neurons [14]. On the other hand, CtBP1 interacts with Hes1 to suppress neurogenesis in the chick dorsal spinal cord [15]. Interestingly, a missense mutation on CtBP1 was found in patients presenting neurodevelopmental deficits (e.g., ataxia, intellectual disability), further supporting the role of CtBP in central nervous system development [16]. Recently, we have demonstrated that CtBP are expressed in subventricular zone neural stem/progenitor cells, and the exposure of neonatal neural stem cell cultures to an unspecific ligand of CtBP, the 4-methylthio 2-oxobutyric acid (MTOB), resulted in enhanced neurogenesis and neuronal complexity as well as increased oligodendrogenesis indicating their potential for regenerative therapies [17].

In the adult mice brain, CtBP1 expression is found in the *substantia nigra* (SN), forebrain, cerebellum, diencephalon, dorsal thalamus, globus pallidus, ventral pallidum, cerebral cortex, and hippocampus, while CtBP2 is highly expressed in the olfactory bulb, cerebellum, cerebral cortex, and hippocampus [18]. Regarding cell survival, it has been shown in epithelial cells and fibroblasts that CtBP promote downregulation of pro-apoptotic genes, including p53 effector related to pmp-22 (PERP), p21, Bax, and Noxa [19–21], while activation of caspase-3, due to ultraviolet irradiation, was more pronounced in *Ctbp1-* and *Ctbp2-null cells* [19, 22]*.* CtBP also seem to promote the survival of cerebellar granule neurons and dopaminergic neuron-like cells *in vitro* [23]. Moreover, the overexpression of CtBP1 in hippocampal and cortical neurons triggers neuroprotection in rat models of Alzheimer’s disease [24]. In Huntington’s disease, the mutant huntingtin (a hallmark of the pathology) showed less affinity to CtBP than its wild-type counterpart in human fibroblasts, which may cause weaker repression of pro-apoptotic genes [25]. Altogether these data points to a relevant role of CtBP in neuronal survival and development, indicating that these proteins could be valuable targets for developing novel therapeutics against neurological disorders such as PD. To date, no studies have assessed the role of CtBP in PD neurodegeneration or aging *in vivo*. In this sense, we hypothesized that CtBP could improve dopaminergic survival in PD models. First, the cellular expression (neurons and glial cells) of CtBP was evaluated in the SN and striatum (ST) of adult mice, the most susceptible regions in PD. Then, CtBP protein levels were assessed in the SN and ST *in vivo* both in physiological conditions (in young adult and old mice) and in experimental models mimicking PD. Then, the effect of CtBP on dopaminergic cell survival was investigated by using a non-specific substrate (MTOB) and siRNAs for each isoform. We first showed the cellular and regional pattern of CtBP expression in the nigrostriatal pathway in healthy young adult and aged mice and in PD preclinical models. Then, we reveal a novel neuroprotective role for CtBP in a 6-OHDA model for PD. Understanding the expression patterns of CtBP in PD neurodegeneration and targeting its function will boost its translation into the clinic.

## Materials and Methods

### N27 Cell Cultures and Cell Treatments

The immortalized rat mesencephalic dopaminergic cell line (N27 cells; a kind gift from Dr. Yoon-Seong Kim, Burnett School of Biomedical Sciences, University of Central Florida) was grown in Roswell Park Memorial Institute (RPMI) 1640 medium (Sigma-Aldrich) containing 2 g/L sodium bicarbonate, 10% fetal bovine serum (FBS; Millipore) and 1 mL/L of penicillin/streptomycin (GIBCO), in a humidified atmosphere of 5% CO_2_ at 37 °C.

Cells were plated at a density of 0.16 × 10^5^ cells *per* cm^2^ in 6-well culture plates (western blot), 0.26 × 10^5^ cells *per* cm^2^ in 48-well (MTT experiments with different 6-OHDA concentrations), or 0.31 × 10^5^ cells *per* cm^2^ in 96-well culture plates (in the remaining MTT experiments). For western blot experiments, N27 cells were exposed to two different 6-OHDA concentrations, 25 μM or 50 μM (Sigma-Aldrich), while in the MTT assay, N27 cells were incubated with different MTOB concentrations (50 μM, 250 μM, 500 μM, 1000 μM or 2500 μM; Sigma-Aldrich) in the presence or absence of 50 μM of 6-OHDA.

For siRNA transfection experiments, cells were plated at a density of 0.21 × 10^5^ cells *per* cm^2^ in 12-well culture plates for western blot or 0.23 × 10^5^ cells *per* cm^2^ in 96-well culture plates for the MTT assay according to our previous protocols [26, 27]. N27 cells were transfected with 35 nM of siRNAs (SMARTpool: siGENOME Ctbp1 siRNA, Dharmacon, catalog number: M-043088–01-0010; SMARTpool: siGENOME Ctbp2 siRNA, Dharmacon, catalog number: M-059787–01-0010) complexed with Lipofectamine RNAiMAX (0.06 µL *per* pmol of siRNA; Invitrogen) for 4 h and maintained in culture for 48 h. Importantly, for the MTT assay, cells were exposed to 50 µM of 6-OHDA and/or 250 µM MTOB for the last 24 h of the experiment.

### MTT Reduction Assay

To assess cell viability, MTT reduction was performed as described previously [27] with some modifications. Briefly, after 24 h of cell treatments, 0.5 mg/mL of MTT (Acros Organics) was added to cells for 4 h at 37 °C. Then, the resultant precipitate was dissolved in 10% SDS and quantified at the wavelength of 570 nm, using a XMark™ Microplate Spectrophotometer (Bio-Rad).

#### *In Vivo* Studies

All mice and rats were bred and handled following institutional, national, and European Community guidelines (2010/63/EU). Young adult (2–4 months old) and aged (26 months old) male C57BL/6 mice, as well as male Wistar rats (8–10 weeks old), were housed in appropriate cages under a controlled environment (12 h light/dark cycles and 22 °C) and with *ad libitum* food and water access.

### PD Models

#### Paraquat (PQ) Rat Model

The chronic administration of PQ was carried out using osmotic minipumps (Alzet Durect, Cupertino, CA) at a dose of 2.5 mg/kg/day with a fluid delivery rate of 0.25 µL/h for a period of four weeks (Alzet model 2004, large pumps) – PQ group. The animals of the control groups were implanted with a minipump filled with sterile saline, the vehicle used to dissolve PQ. The pumps were implanted subcutaneously on the back, slightly posterior to the scapulae (shoulder blades), after rats were anesthetized with intraperitoneal (i.p.) injection of ketamine (90 mg/kg) and xylazine (10 mg/kg). Animals were euthanized 5 weeks after the minipumps were implanted [28].

#### MPTP Mouse Model

MPTP (1-methyl-4-phenyl-1,2,3,6-tetrahydropyridine; Sigma-Aldrich) dissolved in sterile 0.9% NaCl was administrated in adult mice (2–4 month-old) via i.p. in four sessions separated by 2 h intervals. Each individual dose was 15 mg/kg body weight of MPTP in a total of 60 mg/kg. Animals were euthanized 7 days after the MPTP intoxication protocol [29].

#### 6-OHDA Mouse Model and MTOB Treatment

Mice were anesthetized with a mixture of ketamine (90 mg/kg of mouse weight; Imalgene 1000, Merial) and xylazine (10 mg/kg of mouse weight; Rompun 2%, Bayer) i.p.. Then, animals were placed in the digital stereotaxic frame (51,900 Stoelting) and an incision was made in the scalp to expose the skull. MTOB (50 μM – 17 ng – or 250 μM – 85 ng; dissolved in sterile 0.9% NaCl) or saline solution (sterile 0.9% NaCl) were injected in the right SN (Anterior–posterior: -3.0 mm, Medial–lateral: -1.4 mm, Dorso-ventral: -4.4 mm; 2 μL total volume), with a 10 µL Hamilton syringe at a speed of 0.2 μL/min. Some mice were also subjected to a stereotaxic injection of 6-OHDA (10 μg dissolved in 0.1% of ascorbic acid; 2 μL total volume), in the right ST (Anterior–posterior: -0.6 mm, Medial–lateral: -2.0 mm, Dorso-ventral: -3.0 mm) as described previously by us [30, 31]. After the intracerebral injection, the incision was sutured, and mice were kept warm (37 ºC) until they recovered from surgery. Then, the animals were maintained in appropriate cages for 7 days. Four experimental groups were designed: Control; 6-OHDA; 6-OHDA and 50 μM MTOB; 6-OHDA and 250 μM MTOB.

### Tissue Preparation

For western blotting experiments, mice were euthanized by cervical dislocation, while rats were euthanized by i.p. injection of pentobarbital (30 mg/kg). Brains were removed, and the SN and ST were dissected and frozen in liquid nitrogen. The tissue was stored at -80 °C until further processing.

For immunohistochemistry experiments, mice were anesthetized and transcardially perfused with 0.9% NaCl, followed by perfusion with 4% paraformaldehyde (PFA). The brains were collected and further fixed in 4% PFA overnight, followed by soaking in a 30% sucrose solution until sunk. Next, brains were cryopreserved and then coronally sectioned with a thickness of 40 µm using a cryostat (CM 3050S, Leica Microsystems). The slices were kept in an anti-freeze solution (30% ethylene glycol and 30% glycerol in phosphate buffer) until used for immunohistochemistry.

### Immunohistochemistry for Tyrosine Hydroxylase (TH)

To assess dopaminergic neuronal survival, we performed TH-positive (TH^+^) cell counting as described previously [27, 29]. First, the sections were incubated with a blocking solution (0.1% Triton X-100 and 10% FBS in PBS), then the endogenous peroxidases were inhibited with H_2_O_2_ and later incubated overnight at 4 °C with the primary antibody mouse anti-TH (1:500, Transduction Laboratories). After several rinses, the slices were incubated with biotinylated goat anti-mouse secondary antibody (1:200, Vector Laboratories) for 1 h at room temperature. Subsequently, the Vectastain ABC kit was added, and the resulting product was visualized by adding DAB to the slices until color developed (5–10 min). Afterward, the sections were counterstained with Nissl (0.25% Cresyl Violet dissolved in acetate buffer) for 4 min, washed in tap water, air-dried, cleaned with xylene, and mounted with Entellan™ (Merck).

Quantification of the TH^+^ neurons in the mice SN was performed in five consecutive coronal sections separated by 240 µm. The SN was carefully delineated, and the number of TH^+^ cells in each condition was counted. Images were acquired under the magnification of 10 × at the Zeiss Axiovert 200 imaging microscope (Axiobserver Z1, Zeiss), and the number of TH^+^ cells was counted using the ImageJ program.

### Fluorescent Immunostaining

Briefly, brain slices and N27 cells were incubated in a blocking solution (2% FBS and 0.3% Triton X-100 in PBS) for 2 h at room temperature. Brain slices were incubated with the primary antibodies in a blocking solution for 2 overnights (mouse anti-CtBP1,1:1000, BD Biosciences, cat no. 612042; mouse anti-CtBP2, 1:1000, BD Biosciences, cat no. 612044; rat anti-CD11b, 1:1000, Serotec; rabbit anti-GFAP, 1:200, DAKO; rabbit anti-NeuN, 1:500, Cell Signaling; rabbit anti-TH, 1:1000, Santa Cruz Biotechnology), while N27 cells were incubated for 1 overnight (mouse anti-CtBP1 and mouse anti-CtBP2, 1:200) at 4 °C. Afterward, tissue and cells were incubated for 2 h at room temperature with the following appropriated secondary antibodies: Alexa Fluor 594 donkey anti-mouse (Abcam), Alexa Fluor 488 donkey anti-rat (Life Technologies) and Alexa Fluor 647 donkey anti-rabbit (Life Technologies) (1:1000 for tissues and 1:200 for cells). Lastly, sections were rinsed with PBS and mounted in Fluoroshield Mounting Medium (Abcam). Images were acquired under the magnification of 40 × using a Zeiss inverted confocal microscopy (Axiobserver Z1, Zeiss).

### Western Blotting

N27 cells and brain tissue lysates were obtained using RIPA buffer (50 mM Tris, pH = 8.0, 150 mM NaCl, 1% Triton X-100, 0.5% sodium deoxycholate, 0.1% SDS, and a cocktail of protease inhibitors) and mechanical dissociation. Lysates were then centrifuged, and the supernatant was collected and quantified using a Pierce bicinchoninic acid protein assay Kit (Thermo Scientific) according to the manufacturer’s instructions. Then, 40 µg of lysate protein was loaded in a 10% SDS polyacrylamide gel (at 110 V until the samples reached the end of the gel). After electrophoresis and transfer into a polyvinylidene difluoride membrane (1.0 A, 25 V for 25 min at room temperature, using a Trans-blot Turbo System (Bio-Rad)), specific protein bands were detected by using appropriate primary antibodies (mouse anti-CtBP1 and mouse anti-CtBP2, 1:2500; mouse anti-GAPDH, 1:5000, Millipore) and secondary antibodies (goat anti-mouse antibody conjugated with horseradish peroxidase, 1:5000, Santa Cruz Biotechnology) followed by incubation with Luminata Crescendo Western HRP Substrate (Millipore) for 5 min. Protein bands were detected using the ChemiDocTM MP Imaging System (Bio-Rad) and quantified by densitometry analyses using the Image Lab 5.1 software (Bio-Rad Laboratories).

#### Statistical Analysis

All data are expressed as mean ± SEM of at least three independent experiments performed in triplicate (*in vitro*) or at least three different animals (*in vivo)* used for assessing TH-immunoreactivity and CtBP expression levels. Statistical analysis was performed using one-way ANOVA followed by the Dunnett’s or Sidak’s multiple comparisons test or by unpaired two-tailed Student's t-test. Values of *P* < 0.05 were considered significant. Statistical analysis was made using the GraphPad Prism 8.0 Software (GraphPad Sotware Inc.).

## Results

### CtBP1 and CtBP2 Cellular Localization and Expression Levels in the Healthy Adult Mouse Brain

**Fig. 1:**
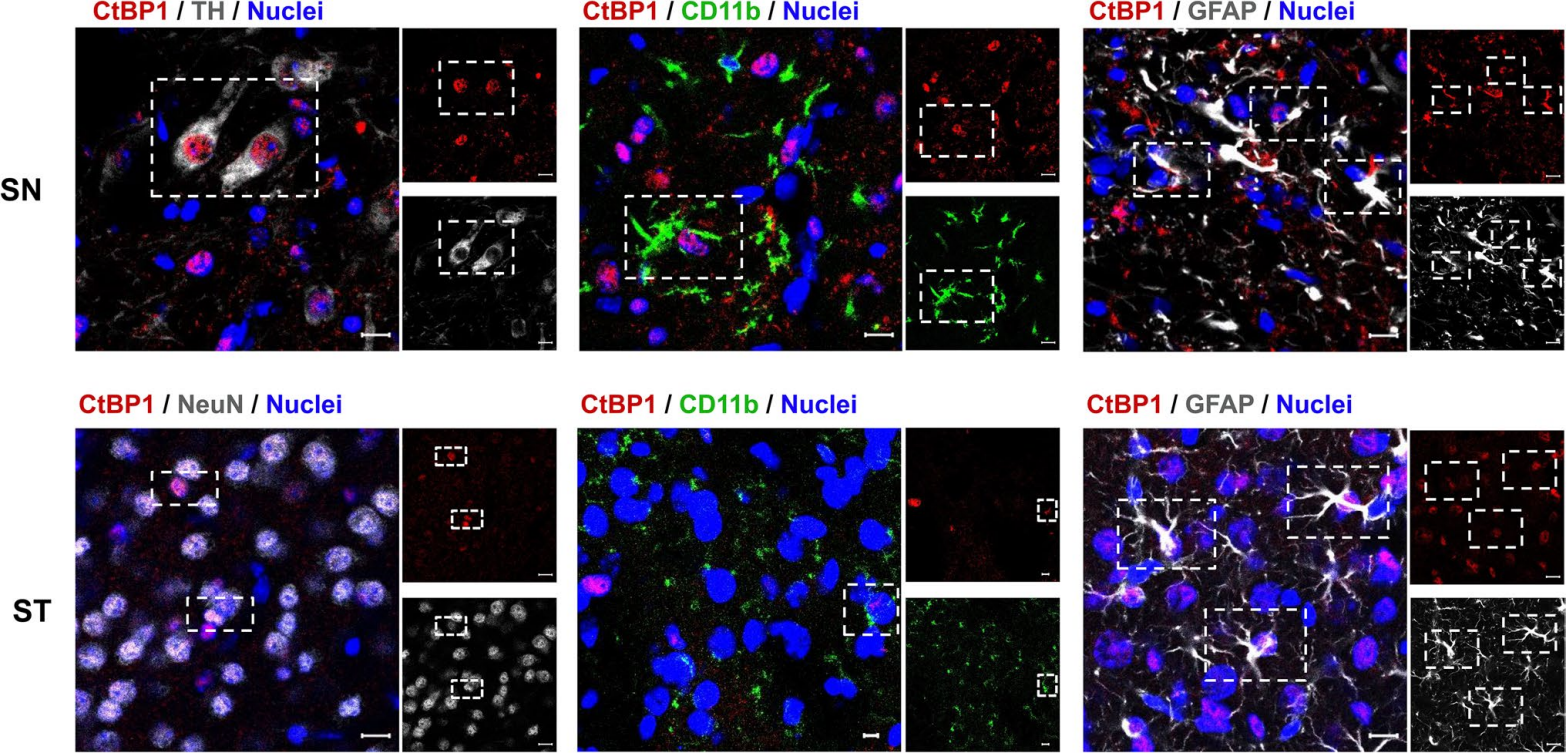
CtBP1 cellular and subcellular localization in the *substantia nigra* and striatum of young adult mice *in vivo. *Representative images of the cellular and subcellular localization of CtBP1 in the *substantia nigra* (SN) and striatum (ST) of young adult mice. CtBP1 (red) is expressed in tyrosine hydroxylase (TH) neurons (dopaminergic marker, top left panel – SN, gray), CD11b cells (microglial marker, middle panels, green), GFAP cells (astrocytic marker, right panels, gray), and mature neurons (NeuN, lower left panel – ST, gray) in both the SN and ST. Nuclei are stained in blue. Dashed rectangles highlight double-positive cells. Scale bar 20 µm

**Fig. 2:**
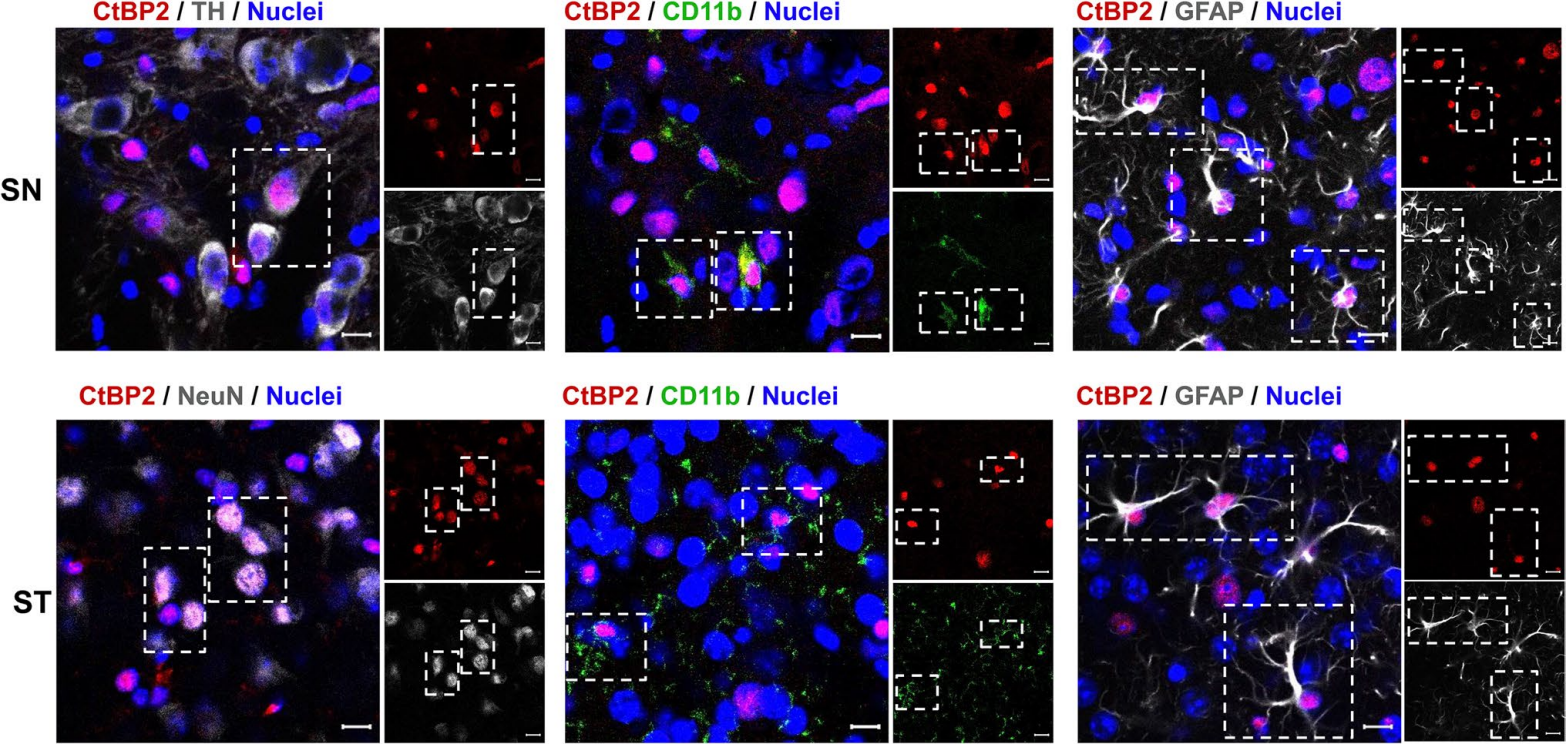
CtBP2 cellular and subcellular localization in the *substantia nigra* and striatum of young adult mice *in vivo. *Representative images of the cellular and subcellular localization of CtBP2 in the *substantia nigra* (SN) and striatum (ST) of young adult mice. CtBP2 (red) is expressed in tyrosine hydroxylase (TH) neurons (dopaminergic marker, top left panel – SN, gray), CD11b cells (microglial marker, middle panels, green), GFAP cells (astrocytic marker, right panels, gray), and mature neurons (NeuN, lower left panel – ST, gray) in both the SN and ST. Nuclei are stained in blue. Dashed rectangles highlight double-positive cells. Scale bar 20 µm

To better understand if CtBP modulation might regulate PD initiation and/or progression, we first performed a comprehensive analysis of the cellular and subcellular localization and expression levels of CtBP1 (Fig. 1) and CtBP2 (Fig. 2) in the SN and ST (most affected areas in PD) of young adult mice. CtBP1 was found to be expressed in all the cell types analyzed in both SN and ST, including dopaminergic neurons (TH^+^ cells; SN), mature neurons (NeuN^+^ cells; ST), microglia (CD11b^+^ cells; SN and ST), and astrocytes (GFAP^+^ cells; SN and ST), as shown in Fig. 1. Not surprisingly, CtBP1 was shown to be majorly expressed in the cell nucleus in both brain regions, although cytosolic expression was also seen. Like CtBP1, CtBP2 was expressed in all the cell types analyzed in SN and ST (dopaminergic neurons, mature neurons, astrocytes, and microglia; Fig. 2). Nevertheless, CtBP2 only displayed nuclear localization.

**Fig. 3:**
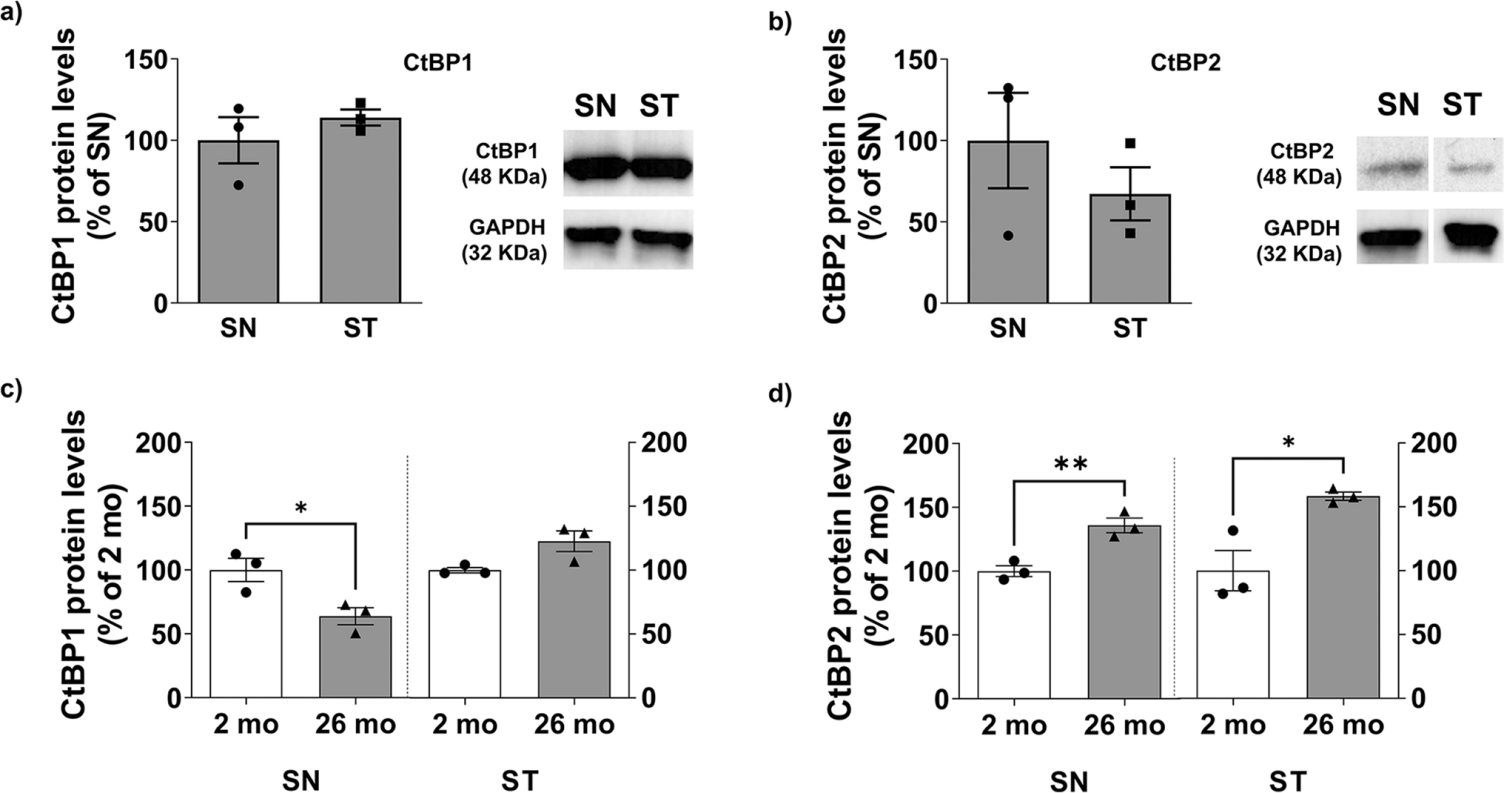
CtBP expression levels in the *substantia nigra* and striatum of young adult and aged mice. Total protein expression levels of CtBP1 **(a)** and CtBP2 **(b)** in SN and ST of young adult mice. On the right of the graph, representative western blotting images against CtBP1 (48 kDa) **(a)** or CtBP2 (48 KDa) **(b)** and GAPDH (36 kDa) are shown. Total protein expression of CtBP1 **(c)** and CtBP2 **(d)** in SN and ST of young adult (2 months old, 2 mo) and aged (26 months old, 26 mo) mice. Data presented as percentage of SN ± SEM, *n* = 3, *P < 0.05 and **P < 0.01 when compared to 2 mo control using the unpaired two tailed Student's t test

The expression levels of CtBP1 and CtBP2 were also quantified in both SN and ST (Fig. 3a, b). Expression levels of CtBP1 in SN and ST were identical (Fig. 3a), while CtBP2 expression in the SN seemed slightly higher than ST, albeit not statistically different (Fig. 3b). Aging is one of the main risk factors in PD development. Therefore, we evaluated CtBP expression levels in the ST and SN of aged mice (26 month-old, 26 mo) and compared them to young adults (2 mo; Fig. 2c, d). Levels of CtBP1 significantly decreased in the SN of aged mice (SN 2 mo = 100.0 ± 9.1%, SN 26 mo = 63.7 ± 6.7%, *n* = 3, *P = 0.0325) but remained unchanged in the ST (Fig. 3c). For CtBP2, both SN and ST levels were increased with age (SN 2 mo = 100.0 ± 4.3%, SN 26 mo = 135.9 ± 5.8%, **P = 0.0075; ST 2 mo = 100.0 ± 15.8%, ST 26 mo = 158.3 ± 3.2%, *P = 0.0224; *n* = 3; Fig. 3d).

### CtBP1 and CtBP2 Expression Levels in PD Rodent Models

**Fig. 4:**
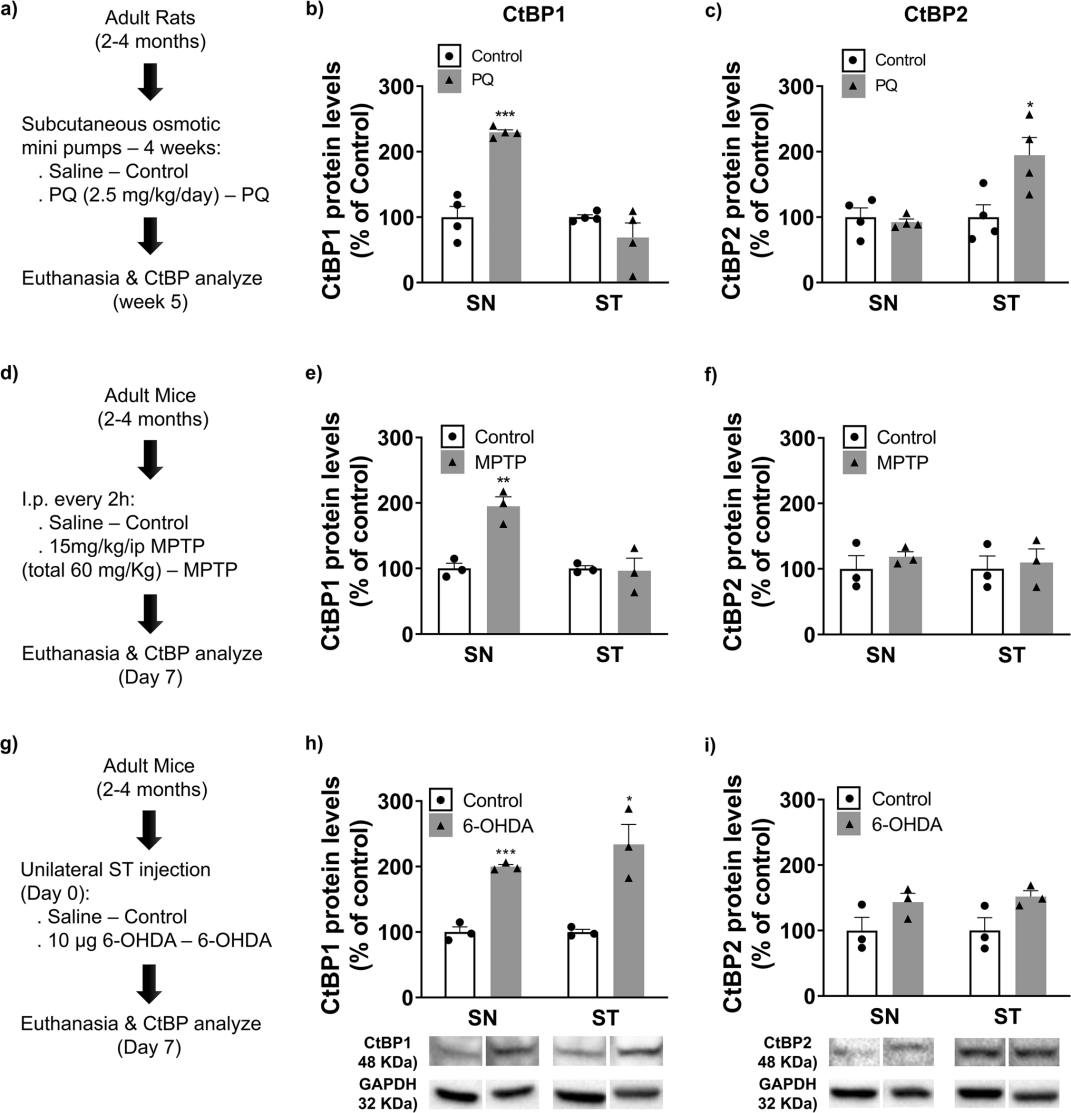
CtBP protein expression levels in rodent models of Parkinson’s disease *in vivo. ***a)** Representative scheme of the paraquat (PQ) rat model experimental setup. Adult rats were administered small dosages of PQ (2.5 mg/kg/day) for 4 weeks through osmotic minipumps implanted in rat’s back, and CtBP analysis was done at week 5. Expression levels of **(b)** CtBP1 and **(c)** CtBP2 in the *substantia nigra* (SN) and striatum (ST) of adult rats challenged with PQ. **d)** Schematic representation of the 1-methyl-4-phenyl-1,2,3,6-tetrahydropyridine (MPTP) model. C57BL/6 adult mice were subjected to intraperitoneal injections (i.p.) of saline (Control) or 15 mg/kg of MPTP every 2 h in a total of 60 mg/kg. Seven days later, mice were euthanized to assess CtBP1 and CtBP2 protein levels in the SN and ST. CtBP1 **(e)** and CtBP2 **(f)** expression levels in the SN and ST of MPTP-challenged mice. **g)** C57BL/6 adult mice were subjected to a unilateral injection in the striatum of either saline (Control) or 6-hydroxydopamine (6-OHDA, 10 µg), and mice euthanized 7 days after the surgery for CtBP regional (SN and ST) expression level analysis. Expression levels of **(h)** CtBP1 and **(i)** CtBP2 in the SN and ST of adult mice challenged with 6-OHDA. Below the graphs, representative western blotting images of CtBP1 (48 kDa), CtBP2 (48 kDa), and GAPDH (36 kDa) are presented. Data are expressed as a percentage of control ± SEM. Protein expression in the control condition was set to 100%. GAPDH was in all the sets for protein normalization. *n* = 3 **(e, f, h, i)** or 4 **(b, c)**, *P < 0.05, **P < 0.01 and ***P < 0.001 when compared to control using the unpaired two tailed Student's t test

Then, to assess a possible correlation between CtBP and PD, we analyzed CtBP1 and CtBP2 protein expression levels in the SN and ST in three PD rodent models (Fig. 4). The paraquat (PQ) model (Fig. 4a) is based on the chronic administration of PQ in adult Wistar rats using osmotic minipumps implanted subcutaneously on the rat’s back. At week 5, animals present alpha-synucleinopathy and approximately 20% reduction in dopamine levels in ST [28]. The MPTP model (Fig. 4d) is based on the i.p. administration of MPTP in C57BL/6 mice. On day 7 after toxin administration, mice show a reduction of TH^+^ cells (around 50%) in the SN and over 70% loss in striatal TH fiber immunoreactivity [29]. Finally, the 6-OHDA model (Fig. 4g) is based on the unilateral local administration of the toxin 6-OHDA into the caudal striatum of mice. The mice show about 50% degeneration of SN dopaminergic neurons 7 days after administration [30, 31]. CtBP1 protein levels were significantly increased in the SN of all 3 rodent models (PQ model, Fig. 4b: Control = 100.0 ± 16.5%, PQ = 229.5 ± 3.8%, *n* = 4, ***P = 0.0003; MPTP model, Fig. 4e: Control = 100.0 ± 8.0%, MPTP = 195.0 ± 14.5%, *n* = 3, **P = 0.0045; 6-OHDA model, Fig. 4h: Control = 100.0 ± 8.0%, 6-OHDA = 199.8 ± 3.2%, *n* = 3, ***P = 0.0003) as well as in the ST of 6-OHDA-challenged mice (Control = 100.0 ± 4.1%, 6-OHDA = 233.8 ± 30.6%, *n* = 3, *P = 0.0122, Fig. 4h) when compared with control mice. On the other hand, CtBP2 protein levels were only significantly higher in the ST of the rat PQ model (Fig. 4c, Control = 100.0 ± 19.0%, PQ = 194.5 ± 27.0%, *n* = 4, *P = 0.0287). Although the levels of CtBP2 were not significantly altered in 6-OHDA treated mice, a similar tendency towards a higher expression, particularly in the ST, was observed (SN: Control = 100.0 ± 20.2%, 6-OHDA = 143.5 ± 13.5%, *n* = 3, *P* = 0.1479; ST: Control = 100.0 ± 19.5%, 6-OHDA = 151.9 ± 9.0%, *n* = 3, *P* = 0.0728; Fig. 4i).

### CtBP1 and CtBP2 Modulation Counteracts Dopaminergic Neuronal Loss in an *In Vitro* 6-OHDA-Induced PD Model

**Fig. 5:**
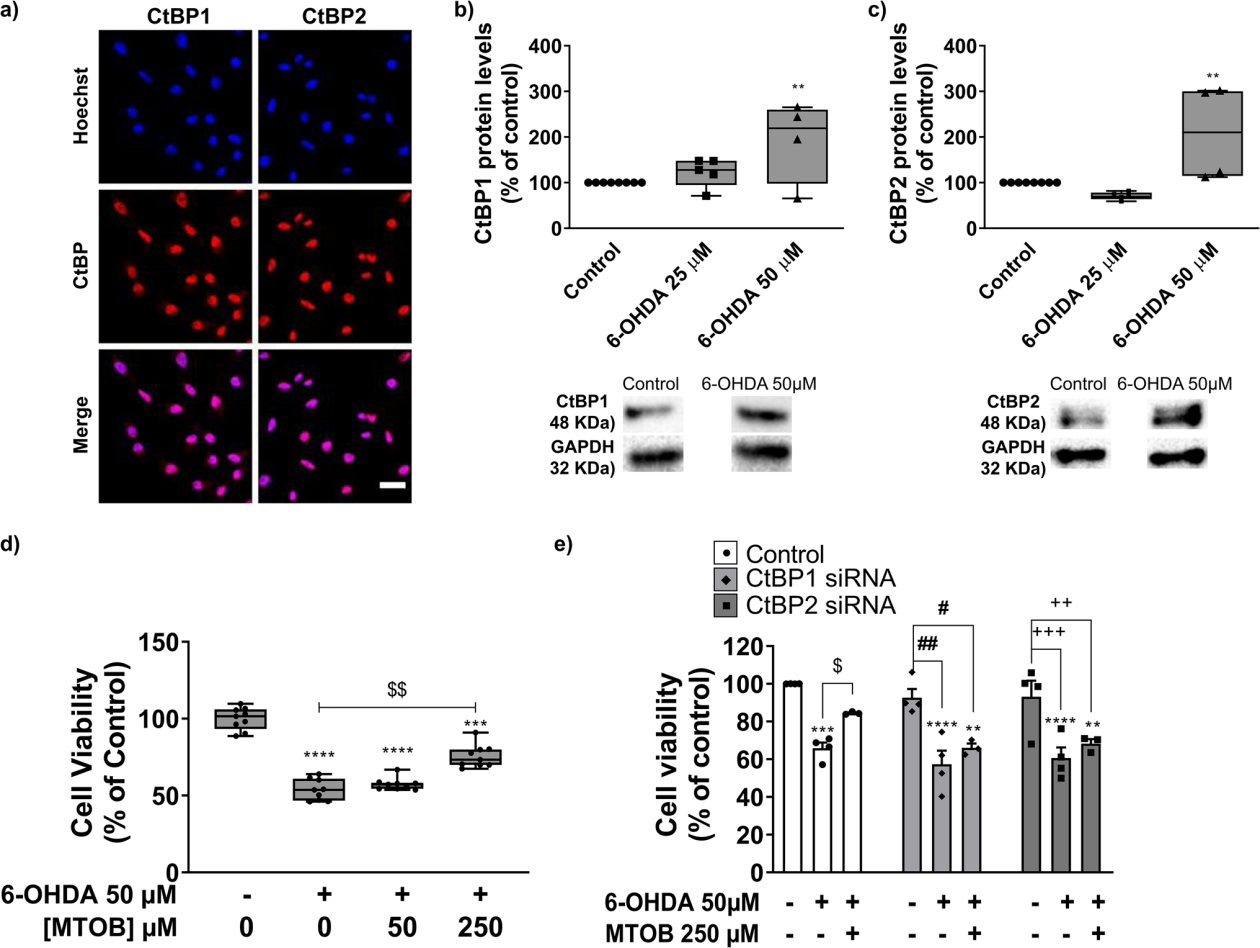
CtBP activation counteracts dopaminergic neurodegeneration in an *in vitro* Parkinson’s disease model.  The dopaminergic cell line N27 was exposed to different dosages of 6-hydroxydopamine (6-OHDA; 25 µM and 50 µM) and/or 4-methylthio 2-oxobutyric acid (MTOB; 50 µM, 250 µM). After 24 h of 6-OHDA and/or MTOB exposure, cell viability was measured by MTT and CtBP expression levels by western blotting. For CtBP inhibition studies, N27 cells were transfected with siRNAs 24 h previous to the 6-OHDA and/or MTOB treatments. **a)** Representative images of the subcellular localization of CtBP1 and CtBP2 proteins (red) in the N27 cells. Nuclei are stained in blue (Hoechst). Scale bar 20 µm. Bar graphs show the expression levels of **(b)** CtBP1 and **(c)** CtBP2 in the N27 cells after treatment with 25 µM or 50 µM of 6-OHDA normalized to GAPDH. Below the graphs, representative images of the western blotting against CtBP1 (48 kDa), CtBP2 (48 kDa), and GAPDH (36 kDa) are presented. **d)** Cell viability after exposure to 50 µM of 6-OHDA with 50 µM or 250 µM of MTOB. **e)** Cell viability of N27 cells without any stimuli, with 50 µM of 6-OHDA alone or in combination with 250 µM of MTOB in basal conditions “Control” or with silencing of CtBP1 “CtBP1 siRNA” or CtBP2 “CtBP2 siRNA”. Data are expressed as a percentage of control ± SEM set to 100%. **(b-d)** *n* = 3–8, **P < 0.01, ***P < 0.001 and ****P < 0.0001 when compared to control and ^$$^*P* < 0.01 when compared to 6-OHDA using the one-way ANOVA, followed by the Dunnett’s multiple comparison test; **(e)** *n* = 3–4, **P < 0.01, ***P < 0.001 and ****P < 0.0001 when compared with Control (without 6-OHDA and MTOB); ^$^*P* < 0.05 vs 6-OHDA; ^#^*P* < 0.05 and ^##^*P* < 0.01 vs CtBP1 siRNA; ^++^*P* < 0.01, ^+++^*P* < 0.001 vs CtBP2 siRNA using one-way ANOVA, followed by the Sidak’s multiple comparison test

Since our data show altered expression levels of CtBP in different PD paradigms, we hypothesized that modulation of CtBP activity could influence dopaminergic survival. In the following experiments, we used the 6-OHDA model only. This model triggered absent or low animal mortality (opposite to PQ and MPTP) and induced local retrograde selective dopaminergic degeneration, closely mimicking PD pathophysiology. We started by testing our hypothesis *in vitro* (Fig. 5 and Supplementary Fig. 1). First, we confirmed the expression of both CtBP1 and CtBP2 in a dopaminergic cell line (N27 cell line). Both CtBP isoforms seem to be expressed in the nucleus, with CtBP1 also being moderately expressed in the cytoplasm (Fig. 5a). Then, the expression levels of CtBP1 (Fig. 5b) and CtBP2 (Fig. 5c) were quantified in non-treated N27 cells (Control) and cultures treated with 6-OHDA (25 µM or 50 µM). These concentrations were chosen because they induced about 20% and 50% reduction in cell viability, respectively (25 µM 6-OHDA = 76.6 ± 4.1%; 50 µM 6-OHDA = 53.7 ± 1.6%, *n* = 5–11, Supplementary Fig. 1a). In accordance with the previous results in Fig. 4, exposure to 50 µM 6-OHDA resulted in a significant increase in terms of CtBP1 and CtBP2 expression (CtBP1: 25 µM 6-OHDA = 122.6 ± 14.1%, 50 µM 6-OHDA = 192.4 ± 44.7%, *n* = 4–5, **P = 0.0087, Fig. 5b; CtBP2: 25 µM 6-OHDA = 70.6 ± 3.7%, 50 µM 6-OHDA = 208.4 ± 52.5, *n* = 4–5, **P = 0.0053, Fig. 5c). Considering that the first motor symptoms of PD usually appear when 30% to 50% of the SN dopaminergic neurons are lost [32], and the altered expression of CtBP at 50 µM of 6-OHDA, this condition was selected for the remaining *in vitro* experiments.

To test if CtBP modulation could protect dopaminergic neurons against 6-OHDA, we then used MTOB. MTOB is a substrate for CtBP dehydrogenase 80 to 5,000-fold more specific than other similar α-ketoacids [33]. Interestingly, MTOB is a substrate of both CtBP, and its catalysis has biphasic kinetics. High MTOB concentrations (millimolar) act as a negative CtBP substrate (inhibition of CtBP function), whereas lower MTOB concentrations act as a CtBP positive substrate (activation of CtBP function) [33]. Nevertheless, to date, only high concentrations of MTOB have been tested in neurons, resulting in cell apoptosis [23]. Therefore, to assess the toxic effect of MTOB *per se* in the N27 cell line, we assess cell viability by testing several MTOB concentrations: 50 μM, 250 μM, 500 μM, 1000 μM, and 2500 μM for 24 h (Supplementary Fig. 1b). Herein, we demonstrate that concentrations above 500 µM are toxic to the cells. Based on these results and the MTOB biphasic kinetics, we selected the concentration of 50 μM and 250 μM (CtBP activation) to test the CtBP neuroprotective potential. We found that 6-OHDA-dependent cell loss was partially reverted in the presence of 250 µM of MTOB (6-OHDA = 53.7 ± 1.6%, 6-OHDA + 50 µM MTOB = 57.4 ± 1.9%, 6-OHDA + 250 µM MTOB = 75.5 ± 4.1%, *n* = 3, ***P = 0.0002, ****P = 0.0001, ^$$^*P* = 0.0024; Fig. 5d).

To investigate if CtBP neuroprotective role depended on the activation of CtBP1 or CtBP2 *per se* or the activation of both simultaneously, we used a pool of CtBP1 or CtBP2 specific siRNA to downregulate their expression in the N27 dopaminergic cell line. Transfection of the cells with the siRNAs against CtBP1 or CtBP2 resulted in around 70% reduction in the levels of each protein in a specific way, 48 h after transfection (Supplementary Fig. 2). Therefore, N27 cells were first transfected with siRNA against CtBP1 or CtBP2 and 24 h later incubated with a combination of 50 µM of 6-OHDA and 250 µM of MTOB, followed by the analysis of cell viability (Fig. 5e). At this experimental setup, 50 µM of 6-OHDA also led to a significant reduction of cell viability in control conditions as well as in the conditions where CtBP1 and CtBP2 was downregulated (Control: 50 µM 6-OHDA = 65.8 ± 3.0%; siRNA α CtBP1: 6-OHDA = 57.3 ± 7.2%, ^##^*P* = 0.0032 vs siRNA α CtBP1 non-treated cells; siRNA α CtBP2: 6-OHDA = 60.6 ± 5.7%, ^+++^*P* = 0.0002, *n* = 4, ***P = 0.0004 and ****P < 0.0001 vs Control non-treated cells; Fig. 5e). However, only in the control conditions (without siRNA) the administration of 250 µM of MTOB was able to partially rescue the 6-OHDA-induced cell death (Control: 6-OHDA + 250 µM MTOB = 84.4 ± 0.4%, ^$^*P* = 0.0204; siRNA α CtBP1: 6-OHDA + 250 µM MTOB = 66.1 ± 2.2%; siRNA α CtBP2: 6-OHDA + 250 µM MTOB = 68.3 ± 2.4%, *n* = 3; Fig. 5e), suggesting that activation of both CtBP is needed to counteract the 6-OHDA toxicity.

### CtBP1 and CtBP2 Modulation Counteracts Dopaminergic Neuronal Loss in 6-OHDA-Challenged Mice *In vivo*

**Fig. 6:**
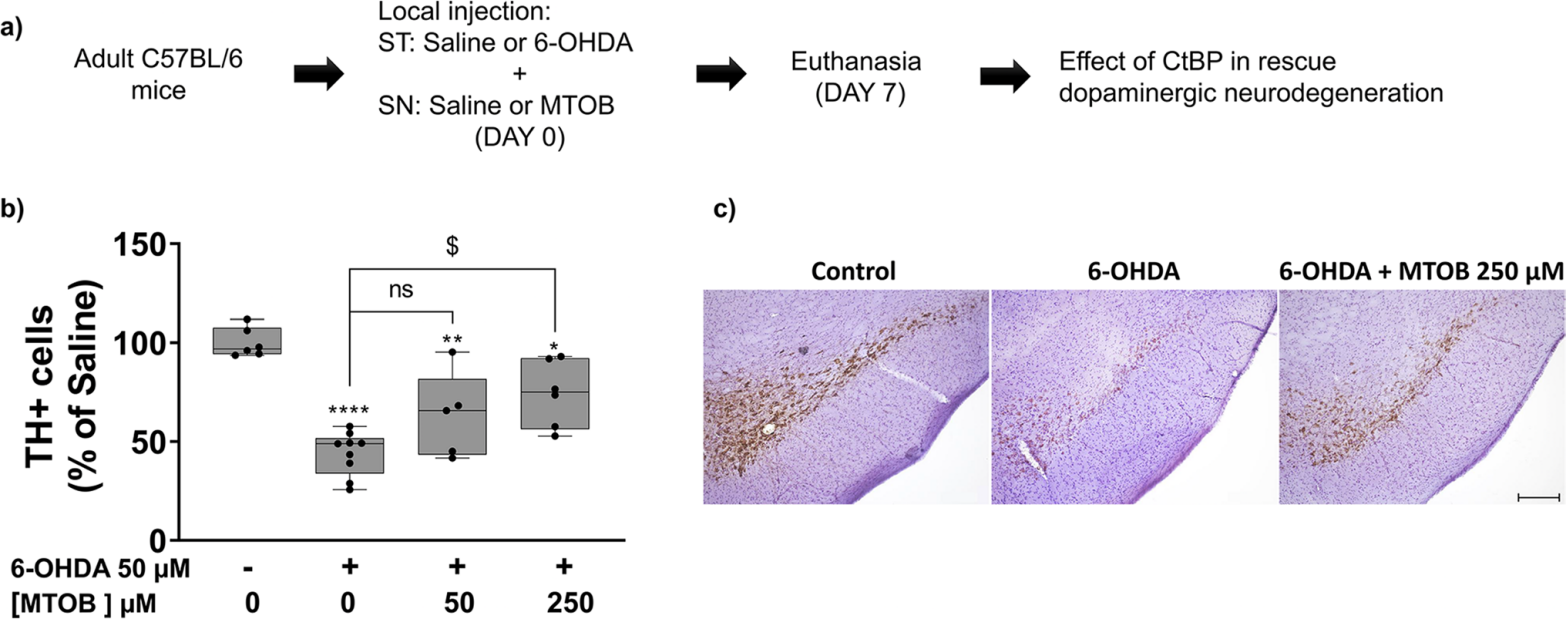
CtBP modulation protects dopaminergic neurons in a 6-OHDA mouse model of Parkinson’s disease. **a**) C57BL/6 mouse were subjected to a double unilateral injection of saline or 6-hydroxydopamine (6-OHDA; 10 µg) in the right striatum (ST) followed by an injection of saline or different dosages of MTOB (50 µM, 250 µM) in the right *substantia nigra* (SN). After 7 days, mice were perfused with 4% paraformaldehyde, and the dopaminergic cell number was assessed by tyrosine hydroxylase (TH) immunohistochemistry. **b)** Bar graph represents the levels of dopaminergic neurons (TH-positive cells; TH^+^) in the SN. Data are expressed as a percentage of control ± SEM. The control condition was set to 100%. *n* = 5–9, *P < 0.05, **P < 0.01 and ****P < 0.0001 when compared to control; ns, non-significant and ^$^*P* < 0.05 when compared with 6-OHDA using the one-way ANOVA, followed by the Dunnett’s multiple comparison test. **c)** Representative images of the dopaminergic neurons in the SN of saline mice, 6-OHDA-exposed mice, and 6-OHDA- and MTOB-exposed mice. TH is shown in brown. Scale bar 200 µm

Our previous *in vitro* data suggest that the modulation of the CtBP activity reveals a neuroprotective role in an *in vitro* PD model. Then, an *in vivo* proof-of-concept was done using the 6-OHDA mice PD model. C57BL/6 mice were subjected to a double unilateral injection of either saline or 10 µg 6-OHDA in the ST, followed by administration of different MTOB concentrations (50 µM, 250 µM) in the SN. Seven days after surgeries, the number of dopaminergic neurons in the SN was counted (Fig. 6a). 6-OHDA caused a reduction of more than 50% in the levels of dopaminergic neurons in the SN (Control = 100.0 ± 3.0%, *n* = 6; 6-OHDA = 42.4 ± 4.4%, *n* = 9, ****P < 0.0001, Fig. 6b, c). In accordance with the results obtained *in vitro* co-administration of 6-OHDA with MTOB at concentrations of 50 µM and 250 µM, tend to decreased 6-OHDA-induced toxicity or even partially reverted it (6-OHDA + 50 µM MTOB = 60.0 ± 11.3%, *n* = 6; 6-OHDA + 250 µM MTOB = 69.9 ± 9.4%, *n* = 5; *P = 0.0197, **P = 0.0031, ^$^*P* = 0.00220; Fig. 6b, c). Altogether, these evidence suggest that CtBP activation protects dopaminergic neurons against degeneration induced by 6-OHDA *in vivo.*

## Discussion

Over the past decades, most information regarding the roles of CtBP1 and CtBP2 focused on embryonic development, oncogenesis, and apoptosis. Overall, studies have shown that CtBP exhibit pro-survival activity in both non-neuronal and neuronal cells [15, 23, 34–36]. Therefore, CtBP emerge as potential targets for treating neurodegenerative diseases. However, few studies to date have explored the role of CtBP in the development and/or progression of neurodegenerative disorders. In particular, no study has focused on the role of CtBP in PD models. Herein, we first explored the subcellular, cellular, and regional expression levels of CtBP isoforms *in vitro* (dopaminergic cell line) and *in vivo* (SN and ST of adult or aged mice) in physiological conditions and toxin-based rodent models of PD. Then, we evaluated the putative neuroprotective effect of CtBP in the 6-OHDA-induced PD model.

Regarding cellular and subcellular expression, we have found that both CtBP were expressed in the neurons, particularly dopaminergic neurons, astrocytes, and microglia both in the ST and SN *in vivo*. CtBP2 was present almost exclusively in the nucleus, while CtBP1 was present in neuronal and glial processes and also in the nucleus. These findings are in line with previous studies suggesting that both isoforms may overlap but also show distinct subcellular locations [9, 18, 37]. Several transcription factors, such as BKLF, can bind CtBP1 and sequester it to the nucleus, acting as a transcriptional corepressor [7]. On the contrary, interaction with neuronal nitric acid synthase (nNOS) and increased neuronal activity [9, 37] directs CtBP1 towards the cytoplasm and presynaptic terminals, thereby hindering the function of CtBP1 as a corepressor. In the cytoplasm, CtBP play essential roles, such as in Golgi fission and membrane fission [38] and in the regulation of presynaptic neurotransmitter release and synaptic plasticity in neurons [9, 10]. Our study contributes to a clearer understanding of the cellular and subcellular expression of both isoforms, particularly in the SN and ST, the most vulnerable regions in PD.

Expression levels of CtBP were also analyzed in the SN and ST of healthy (young adult and aged mice) and toxin-injured animals. In the brains of 26-month-old mice (i.e. aged mice), a significant decrease in CtBP1 expression in the SN and a significant increase of CtBP2 expression in the SN and ST were observed. These data suggest that CtBP1 and CtBP2 proteins might have non-overlapping roles in the aged brain. Downregulation of CtBP1 and CtBP2 in a proteasomal- or caspase-dependent way was observed in apoptotic neurons [23]. Interestingly, studies in *Caenorhabditis elegans* showed that CtBP1 is expressed in their central nervous system through development and adulthood, and its downregulation prolongs the lifespan of these nematodes [39, 40]. Therefore, lower levels of CtBP1 in SN may represent a protection mechanism for the dopaminergic neurons that populate the SN and project their fibers into the dorsal ST, which are more susceptible to degeneration or result from neurodegeneration occurring during aging. So far, there is no information about the role of CtBP2 in brain aging, so the interpretation of these data warrants additional experiments that are out of this manuscript's scope. Still, these data are important to open new avenues for studying the role of CtBP in aging.

In all the rodent models of PD assessed (i.e. PQ, MPTP, and 6-OHDA), a significant increase in CtBP1 levels in the SN was observed. Contrary to those observed in aged brains, this increase in PD models suggests a compensatory response to dopaminergic neuronal injury. CtBP1 levels were only augmented in the ST of 6-OHDA-challenged mice, justified probably by the local administration compared with the systemic administration paradigm of the PQ and MPTP models. This observation might also indicate that CtBP display a lower need for compensatory mechanisms in the ST than the SN, which may reflect the mechanisms and extent of neurodegeneration. CtBP2 levels remained unchanged in both SN and ST of MPTP and 6-OHDA mice models, except in the ST of PQ-based rat model, which was found increased. PQ-challenged animals are the only rat model, and they also have a different administration paradigm (chronic) compared to the other two toxins (acute). This model represents an earlier stage of human PD pathology. At week 5, it presents only mild neurodegeneration signals, including alpha-synuclein pathology and mild reduction in striatal dopamine levels [28]. Additionally, PQ is highly toxic and can cause peripheral organ damage, such as liver, kidney, and lung, which induces high lethality in this model [41, 42]. The overall central and peripheral toxicity induced by PQ may explain the increased expression of both CtBP1 and CtBP2. Another fact one cannot exclude is differences related to the species [43]. Regarding the *in vitro* PD model, we have found increased CtBP1 and CtBP2 expression in the rat dopaminergic cell line N27 exposed to 6-OHDA. Interestingly, Stankiewic and collaborators showed that 6-OHDA induced a decrease in CtBP expression in N27 cells *in vitro*. Stankiewic et al. analyzed both alive and dead N27 cells after exposure to the 6-OHDA showing a significant decrease of CtBP1 and 2 in compromised N27 cells [23]. In the present study, we only consider cells that survived the 6-OHDA treatment, which may explain the contradictory results. Moreover, our *in vitro* data were also corroborated by *in vivo* analysis in the SN and ST lesioned regions. Altogether, the increased CtBP expression found in our PD models may suggest a compensatory response to dopaminergic neuronal injury, which seems more specific for CtBP1. Others have reported compensatory mechanisms that occur in the pre-symptomatic stages of the disease, including increased dopaminergic activity, enhanced TH activity and dopamine release in the ST, elevated levels of antioxidant enzymes, among others [44, 45]. The pathophysiology and impact of these compensation mechanisms in the progression of the disease remain unclear and poorly investigated. Importantly, CtBP act as metabolic and redox sensors and react upon neuronal activity [9], suggesting its involvement in expression and/or activity in those earlier events. However, a correlation between the metabolic and redox state of the cells and CtBP expression/activity has not been definitively established in the context of neurodegenerative diseases and warrants further investigation. Understanding these mechanisms could lead to the development of disease-modifying therapeutic approaches and should be investigated in the future.

Next, we evaluated the effects of CtBP on dopaminergic survival by using MTOB, a substrate of both CtBP, which so far presents the highest affinity to CtBP due to the presence of a tryptophan residue in CtBP active site, unique to this dehydrogenase [33, 46]. It acts as an inhibitor for CtBP at high concentrations (millimolar range), inhibiting the recruitment of CtBP to several target promoters, including pro-apoptotic promoters, and, therefore, decreasing the survival of several tumor cell lines and tumor growth *in vivo* [35, 36]. So far, MTOB was used in brain cells mainly at high concentrations (1–5 mM), leading to apoptosis [17, 23]. In a previous report, we showed that low levels of MTOB (up 100 µM) were not toxic to neural stem cell cultures and promoted neuronal differentiation and maturation, and oligodendrogenesis [17], suggesting that CtBP may co-activate genes beneficial for brain regeneration. Herein, we observed a toxic effect of MTOB *per se* at high concentrations, namely at 1000 µM and 2500 µM. Conversely, MTOB was able to counteract 6-OHDA-induced cell death at low concentrations (50 µM to 250 µM). MTOB at low concentrations can act as a CtBP substrate, as described previously [33], leading to a rapid increase in cell viability, probably by repressing some pro-apoptotic genes [19, 21, 36, 47]. For example, Kim and collaborators have shown that CtBP knockout increases Bax transcription and reduces mitochondrial activities in fibroblast cell lines, suggesting that CtBP are important to maintain mitochondrial activities [21], which is also relevant in the context of PD. In another study, the glycolytic inhibitor, 2-deoxy-D-glucose, suppressed seizure activity in a model of temporal lobe epilepsy via an NRSF/CtBP-dependent repression of the brain-derived neurotrophic factor gene promoter [48]. Recently, it was also shown that overexpression of CtBP1 in Alzheimer’s disease rat models induced neuroprotection of hippocampal and cortical neurons and enhanced neuronal activity [24]. Our study supports a neuroprotective role for CtBP in PD, which is corroborated by these previous studies demonstrating that CtBP are key factors for neuronal survival and brain function. Results obtained with low MTOB concentrations were confirmed by CtBP silencing with siRNA, which caused a loss of the neuroprotective effect achieved with these MTOB concentrations. CtBP isoforms have specific but also overlapping functions [3]. Our silencing data obtained *in vitro* suggest that in these experimental conditions, both isoforms act redundantly, both contributing to the survival of dopaminergic neurons. Nevertheless, one cannot exclude some MTOB effects in a CtBP-independent fashion since it can also interact with other proteins and is part of the methionine salvage pathway. Besides the putative direct effects observed in dopaminergic neurons, we cannot exclude an indirect effect on glial cells *in vivo* since CtBP1 and CtBP2 are expressed in microglia cells and astrocytes in the ST and SN of healthy mice. These cells participate in immune responses, and both CtBP have been reported to promote the expression of pro-inflammatory genes in primary microglia and astrocyte cultures upon lipopolysaccharide activation [49]. Moreover, Saijo et al. demonstrated that 5-androsten-3β,17β-diol (ADIOL) mediates the recruitment of CtBP to the promoter’s region of inflammatory responsive genes together with c-Jun/c-Fos AP1-heterodimers, repressing the transcription of these genes [50]. In our paradigm, relatively low MTOB concentrations may also recruit CtBP to repress pro-inflammatory genes and prevent an inflammatory response by microglia and astrocytes. Thus, enhancing CtBP function in both neurons and glial cells may offer a novel and largely unexplored therapeutic target for treating neurodegenerative diseases. This putative anti-inflammatory role of CtBP in PD should be explored in future studies to further evolve into its clinical application in neurodegenerative diseases. In fact, it has been used in the clinic to treat uremic patients [51, 52]. So, better knowledge of the expression and function of CtBP in neurodegenerative conditions is essential to better understand the pathophysiology, identify novel therapeutic targets, and advance into clinical translation.

## Conclusions

CtBP are expressed in neurons, dopaminergic neurons, astrocytes, and microglia cells in the SN and ST of wild-type adult mice. CtBP showed distinct expression patterns in the SN and ST of young adult, aged, and injured animals. CtBP1 expression in the SN is decreased in aged animals and increased in all toxin-injured animals compared with healthy adult animals. CtBP2 is increased in aged animals in both SN and ST and in the ST of PQ-intoxicated animals. *In vitro*, both CtBP isoforms showed increased expression in N27 cells treated with 6-OHDA. N27 cells treated with MTOB showed a dual effect: high concentrations inhibited CtBP and induced cell death while low concentrations acted as an agonist, counteracting 6-OHDA-induced cell death. The protective effect mediated by low concentrations of MTOB was confirmed by CtBP siRNA silencing, which led to a loss of the ability to stimulate these proteins and, consequently, hindered MTOB's ability to protect against 6-OHDA-induced cell death. Thus, synthesizing and/or optimizing drugs able to regulate CtBP activity, such as MTOB, could be potentially explored for developing future therapies for PD patients. However, further detailed investigation of the mechanisms mediated by CtBP in PD models, including genetic PD models and human-derived models, and its clinical usefulness is warranted. In sum, our study demonstrates that CtBP are relevant targets for toxin-based PD models, which might translate into future therapeutic targets in PD and other neurodegenerative diseases.

## Supplementary Information

Below is the link to the electronic supplementary material.

Supplementary file1 (DOCX 471 KB)

## Data Availability

The datasets used and/or analyzed during the current study are available from the corresponding author on request.

## Abbreviations

6-OHDA: 6-Hydroxydopamine
ADIOL: 5-Androsten-3β,17β-diol
AP1: Activating protein 1
Bax: Bcl-2-associated X protein
BKLF: Basic Krüppel-like factor
CD11b: Cluster of differentiation 11b
CtBP: C-terminal binding proteins
FBS: Fetal bovine serum
GAPDH: Glyceraldehyde-3-phosphate dehydrogenase
GFAP: Glial fibrillary acidic protein
i.p.: Intraperitoneal
MPTP: 1-Methyl-4-phenyl-1,2,3,6-tetrahydropyridine
MTOB: 4-Methylthio 2-oxobutyric acid
MTT: 3-[4,5-Dimethylthiazol-2-yl]-2,5 diphenyl tetrazolium bromide
NAD: Nicotinamide adenine dinucleotide
NeuN: Neuronal nuclear protein
nNOS: Neuronal nitric oxide synthase
Noxa: Phorbol-12-myristate-13-acetate-induced protein 1
NRSF: Neuron-restrictive silencer factor
PERP: P53 effector related to pmp-22
PFA: Paraformaldehyde
PD: Parkinson’s disease
PQ: Paraquat
PXDLS motif: Pro-X-Asp-Leu-Ser motif
RRT domain: Arg-Arg-Thr domain
SN: *substantia nigra*
ST: Striatum
TH: Tyrosine hydroxylase
