# Supplementary material for: CtBP Neuroprotective Role in Toxin-Based Parkinson’s Disease Models: From Expression Pattern to Dopaminergic Survival
Source: Mol Neurobiol. 2023 Apr 15;60(8):4246–60. doi: 10.1007/s12035-023-03331-w (PMC10293336; doi:10.1007/s12035-023-03331-w)
Supplement: Supplementary file 1 — Supplementary file1 (DOCX 471 KB) [file 12035_2023_3331_MOESM1_ESM.docx]

**Supplementary Information**

**CtBP neuroprotective role in toxin-based Parkinson’s disease models: from expression pattern to dopaminergic survival.**

**Cláudia Saraiva^1,#^, Jéssica Lopes-Nunes^1^, Marta Esteves^1^, Tiago Santos^1^, Ana Vale^1^, Ana Clara Cristóvão^1^, Raquel Ferreira^1,$^, Liliana Bernardino^1,**^**

^1^Health Sciences Research Centre (CICS-UBI), Faculty of Health Sciences, University of Beira Interior, Covilhã, Portugal

^#^current affiliation: Luxembourg Centre for Systems Biomedicine (LCSB), University of Luxembourg, 7 avenue des Hauts-Fourneaux, Esch-sur-Alzette, Luxembourg

^$^Current affiliation: CEDOC, NOVA Medical School|Faculdade de Ciências Médicas, Universidade NOVA de Lisboa, Campo dos Mártires da Pátria, 130, Portugal

E-mail addresses: CS: [c.m.m.saraiva@gmail.com](mailto:c.m.m.saraiva@gmail.com); JLN: [jessicalonu@hotmail.com](mailto:jessicalonu@hotmail.com); ME: [mrc.esteves@gmail.com](mailto:mrc.esteves@gmail.com); TS: [tiagodesousasantos@gmail.com](mailto:tiagodesousasantos@gmail.com); AV: [ana.vale@ubi.pt](mailto:ana.vale@ubi.pt); ACC: [aclara@fcsaude.ubi.pt](mailto:aclara@fcsaude.ubi.pt); RF: [rql@ubi.pt](mailto:rql@ubi.pt); LB: [libernardino@fcsaude.ubi.pt](mailto:libernardino@fcsaude.ubi.pt)

**^**^Corresponding author:**

Liliana Bernardino, Brain Repair Group, Health Sciences Research Center (CICS-UBI), University of Beira Interior, Av. Infante D. Henrique, 6200-506 Covilhã – Portugal

[libernardino@fcsaude.ubi.pt](mailto:libernardino@fcsaude.ubi.pt)


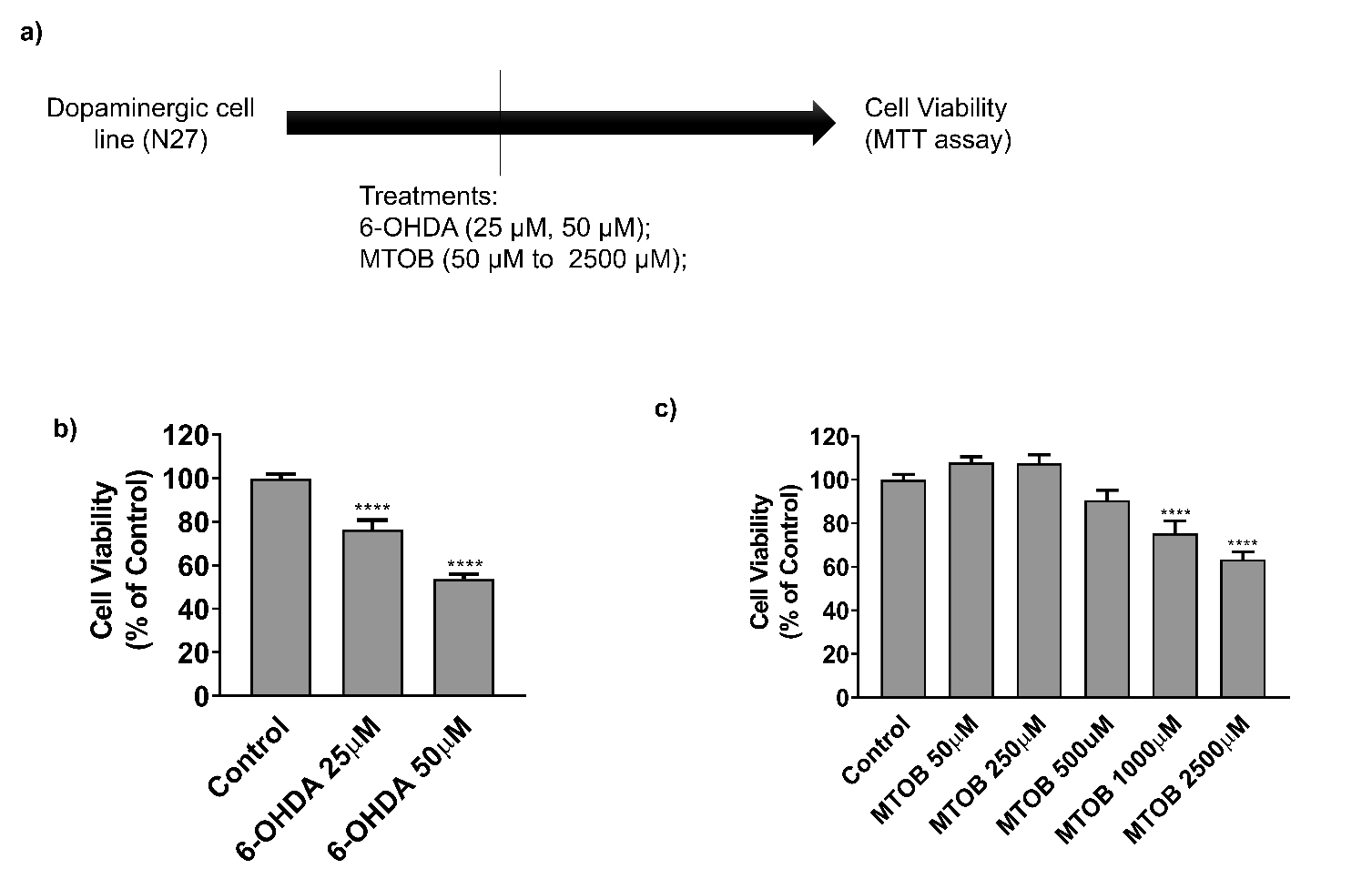
**Supplementary Figure 1: Effects of 6-OHDA and MTOB in the survival of dopaminergic neurons in an *in vitro* model of Parkinson’s disease (PD).**

Dopaminergic cell line (N27 cell line) was exposed to different dosages of the PD-inducer toxin 6-hydroxydopamine (6-OHDA; 25 µM and 50 µM **(a)** and/or different dosages of the unspecific ligand of CtBP, the 4-methylthio 2-oxobutyric acid (MTOB; 50 µM, 250 µM, 500 µM, 1000 µM, 2500 µM **(b)**. After 24 h of cell treatment, cell viability was measured by MTT. Bar graphs represent cell viability after exposure to 6-OHDA **(a)** and MTOB **(b)**. Data are expressed as a percentage of control ± SEM. The control condition was set to 100%. N = 3-11, ****P < 0.0001 when compared to control using the one-way ANOVA, followed by Dunnett’s multiple comparison test.


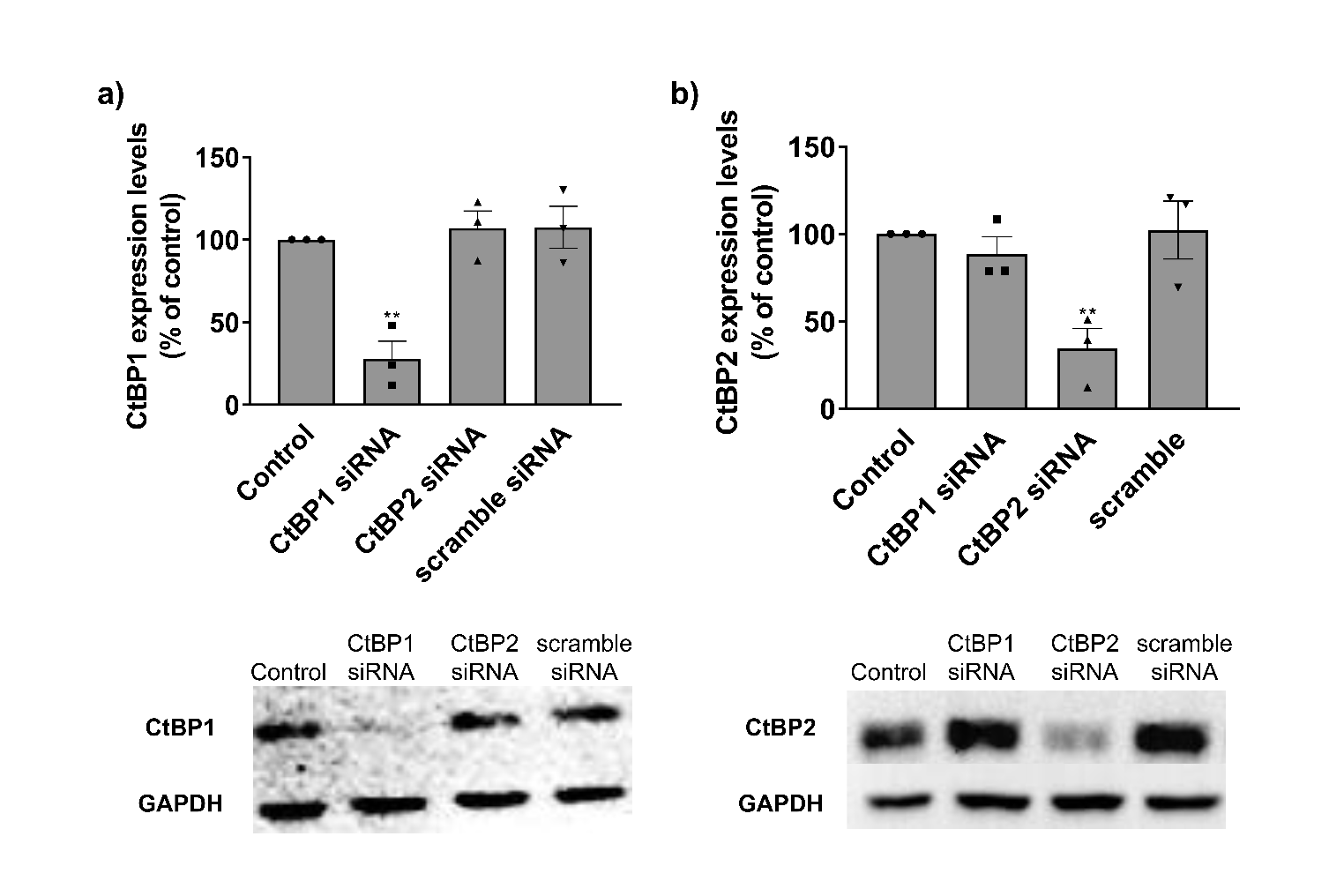
**Supplementary Figure 2: CtBP knockdown efficiency.**

Bar graphs depict the percentage of **(a)** CtBP1 and **(b)** CtBP2 in N27 cells transfected with 35 nM of siRNAs (against CtBP1, CtBP2 or scramble) using lipofectamine RNAiMAx. Protein expression was normalized to GAPDH. Bellow the graphs, are depicted western-blotting representative images of CtBP1 (48 kDa), CtBP2 (48 kDa) and GAPDH (36 kDa). Data are expressed as a percentage of mean ± SEM (n = 3). Protein expression in the control condition was set to 100%. **P < 0.01 vs control using one-way ANOVA, followed by Dunnett’s multiple comparison test.
